# Facilitators and Barriers to Effective Implementation of Interprofessional Care for Type 2 Diabetes in the Elderly Population of the Southern Africa Development Community: A Systematic Review

**DOI:** 10.3390/ijerph22091334

**Published:** 2025-08-27

**Authors:** Ushotanefe Useh, Bashir Bello, Abdullahi Adejare, Koketso Matlakala, Evans Mohlatlole, Olebogeng Tladi

**Affiliations:** 1 Lifestyle Diseases Research Entity, Faculty of Health Sciences, North-West University, Mafikeng 2790, South Africa; ushotanefe.useh@nwu.ac.za (U.U.); 40517357@nwu.ac.za (B.B.); koketso.matlakala@nwu.ac.za (K.M.); evans.mohlatlole@nwu.ac.za (E.M.); olebogengtladi@nwu.ac.za (O.T.); 2 Physiotherapy Department, Bayero University, Kano 700006, Nigeria; 3 Department of Physiology, Faculty of Basic Medical Sciences, College of Medicine, University of Lagos, Lagos 101017, Nigeria; 4 Department of Social Work, North-West University, Mafikeng 2790, South Africa

**Keywords:** diabetes mellitus type 2 (T2D), elderly population, interprofessional care, systematic review, Southern Africa Development Community (SADC)

## Abstract

Background: The management of older diabetic patients in the Southern Africa Development Community (SADC) has been described by several authors as poor due to several constraints and lack of a team care approach. This systematic review aimed to investigate the facilitators and barriers to the effective implementation of interprofessional care (IPC) of the elderly with type 2 diabetes mellitus (T2D) in the SADC region. Methods: A comprehensive literature search was conducted using the Population–Concept–Context (PCC) framework in the search for relevant articles. Out of a total of 155 relevant articles, only 8 articles matched the set criteria and were selected for the final review. Preferred Reporting Items for Systematic Reviews and Meta-Analyses guidelines were used in the review. Results: The identified facilitators include providing decision support to healthcare workers, training of healthcare workers, use of local languages during the training sessions, and use of certified guidelines in the management of not only T2D but also all the other disease conditions. Barriers like ill-equipped patients with limited opportunities for education and counseling, enormous workload due to staff shortages, and loss to follow-up, among others, were equally identified. Conclusions: This systematic review identifies key facilitators and barriers to implementing effective interprofessional care for type 2 diabetes management in the elderly population of the SADC. Understanding these factors can help healthcare professionals optimize their collaborative efforts, ultimately enhancing the quality of care and improving health outcomes for elderly patients with T2D in the region.

## 1. Introduction

Type 2 diabetes (T2D) is an escalating public health concern globally, with the burden particularly rising among older adults [1]. By 2030, 643 million, and by 2045, 783 million older adults are projected to be living with diabetes. Thus, while the world’s population is estimated to grow 20% over this period, the number with diabetes is estimated to increase by 46% [2] with the majority residing in low- and middle-income countries. In sub-Saharan Africa, including the Southern African Development Community (SADC), a regional bloc of 16 member countries [3], diabetes prevalence is rapidly increasing due to urbanization, changing dietary patterns, sedentary lifestyles, and population aging [4,5]. This trend places enormous strain on already under-resourced health systems and calls for innovative, scalable approaches to chronic disease management. Elderly individuals with T2D face complex care needs due to the interplay of aging, multimorbidity, frailty, cognitive decline, and limited social support [6]. Effective care for this population requires more than biomedical treatment; it demands coordinated attention to functional, emotional, nutritional, and social health. However, traditional care models in many SADC countries remain fragmented, provider-centered, and poorly equipped to address the multifaceted nature of diabetes management in older adults [7].

Interprofessional care (IPC) has emerged as a promising strategy to bridge these gaps in care. IPC is defined as the collaborative practice of professionals from multiple disciplines working with patients, families, and communities to deliver comprehensive, person-centered health services [8]. Reeves et al. [9] explained that IPC integrates the expertise of physicians, nurses, pharmacists, dietitians, physiotherapists, social workers, and community health workers to improve health outcomes and the quality of life for patients with chronic conditions like T2D. While the effectiveness of IPC in improving diabetes outcomes has been demonstrated in high-income settings [10,11], the implementation of IPC in resource-constrained environments such as the SADC remains a significant challenge. Limited infrastructure, shortage of skilled health workers, inadequate training in collaborative practices, poor inter-sectoral coordination, and underfunded health systems all hinder the full operationalization of IPC in this region [12,13]. Moreover, the success of IPC is heavily context-dependent, relying on factors such as leadership, organizational culture, role clarity, and policy support, all of which vary significantly across the diverse healthcare landscapes within the SADC. For example, while South Africa has made strides in implementing ward-based outreach teams (WBOTs) composed of multidisciplinary members under its re-engineered primary healthcare model, other SADC countries lag behind in formalizing interprofessional practice structures [14]. Furthermore, IPC implementation in the SADC region may encounter sociocultural barriers such as professional hierarchies, lack of mutual respect between cadres, resistance to role flexibility, and limited community awareness of team-based care models. Language barriers, traditional health beliefs, and poor digital infrastructure also limit effective communication and collaboration among healthcare teams, especially in rural settings [15].

Despite these challenges, several enablers of IPC implementation in the region have been identified. Facilitators include supportive government policies, investment in continuing professional education, institutional champions for interprofessional practice, community engagement, and integration of IPC principles into undergraduate curricula [8,13]. In some contexts, donor-funded programs have also played a catalytic role in introducing team-based models of care, particularly in the management of HIV/AIDS and maternal–child health, which could inform diabetes-related IPC models. Crucially, much of the literature on IPC in the SADC has focused on infectious diseases or general primary healthcare. There is a paucity of synthesized evidence specifically addressing the implementation of IPC for T2D among the elderly. This gap is particularly important given that elderly individuals face additional vulnerabilities such as physical disability, polypharmacy, transportation limitations, and social isolation, which could complicate care coordination and underscore the need for a well-functioning IPC model [15]. Importantly, due to the distinct genetic makeup of SADC residents, urbanization, limited inclusion in global research, the region’s unique T2D pathophysiology, and the substantial number of undiagnosed cases particularly among older adults, without focused consideration for this demographic, IPC interventions may be insufficiently customized or ineffectively implemented [16]. Moreover, implementation science emphasizes the need to understand not only what works but also how and why interventions succeed or fail in real-world contexts. Identifying the barriers and facilitators to IPC implementation in SADC countries is critical for adapting global best practices to local realities. Factors such as political will, workforce capacity, institutional leadership, interprofessional training, community readiness, and funding mechanisms are central to sustainable IPC integration but remain poorly explored in the region.

This systematic review sought to answer a critical research question: what are the facilitators and barriers to effective implementation of interprofessional care approaches in the management of T2D within the SADC region? A systematic review of this nature is necessary to inform regional policy and practice by synthesizing existing evidence on the facilitators and barriers to effective IPC implementation in managing T2D among older adults in SADC. This review will be the first of its kind to focus specifically on the facilitators and barriers to IPC implementation, particularly in the older population within the region. By mapping the contextual and structural factors that influence IPC uptake, this review will highlight key lessons for scaling up team-based care and improving outcomes for older adults with diabetes. Additionally, the findings may contribute to broader discussions on strengthening primary healthcare systems, achieving universal health coverage, and addressing the needs of aging populations in sub-Saharan Africa.

## 2. Materials and Methods

### 2.1. Study Design

This study was conducted as a systematic review guided by the Preferred Reporting Items for Systematic Reviews and Meta-Analyses (PRISMA) 2020 guidelines [17]. The review aimed to synthesize available evidence on the facilitators and barriers to effective IPC for managing type 2 diabetes (T2D) in the elderly population within the SADC region.

### 2.2. Eligibility Criteria

To ensure a focused and relevant synthesis, eligibility criteria were defined using the Population–Concept–Context (PCC) framework. Eligible studies included primary research articles, both the peer-reviewed and gray literature, that examined IPC interventions directed at elderly individuals (aged 60 years and above) with T2D in any SADC country. Studies had to explicitly report on facilitators and/or barriers to IPC implementation. Both qualitative and quantitative designs were included, such as observational studies, randomized controlled trials, implementation studies, and mixed-methods research. Studies not conducted within the SADC context, those not involving IPC, and those not targeting the elderly population with T2D were excluded. Additionally, reviews, editorials, letters, and protocols without findings were omitted. The inclusion and exclusion criteria are as presented in Table 1.

### 2.3. Information Sources

A comprehensive search was conducted across multiple databases and gray literature sources. The following databases were searched: PubMed/MEDLINE, Scopus, Web of Science, Embase, CINAHL, and African Journals Online (AJOL) [18]. For the gray literature, searches were extended to Google Scholar, the WHO Institutional Repository for Information Sharing (IRIS), and websites of Ministries of Health from SADC member states. Reference lists of eligible studies and relevant reviews were also manually screened for additional sources. At the end of the search, we identified 155 articles (PubMed = 20; Scopus = 15; Google Scholar = 95; Web of Science = 15; other sources = 10).

### 2.4. Search Strategy

The search strategy was developed using a combination of controlled vocabulary and free-text terms. Key search terms included combinations of “interprofessional care”, “collaborative practice”, “type 2 diabetes”, “elderly”, “older adults”, “implementation”, “barriers”, “facilitators”, and “Southern African Development Community”, along with individual country names. Boolean operators were applied to ensure comprehensive retrieval of records. Searches were restricted to studies published between January 2000 and June 2025. An example of a PubMed search string was as follows:

(“interprofessional care” OR “multidisciplinary” OR “team-based care”) AND (“type 2 diabetes” OR “T2DM”) AND (“elderly” OR “older adults”) AND (“barriers” OR “facilitators”) AND (“Southern African Development Community” OR “SADC” OR [list of SADC countries]). See Appendix A for a full description of the search process of one of the databases.

### 2.5. Study Selection

**Figure 1:**
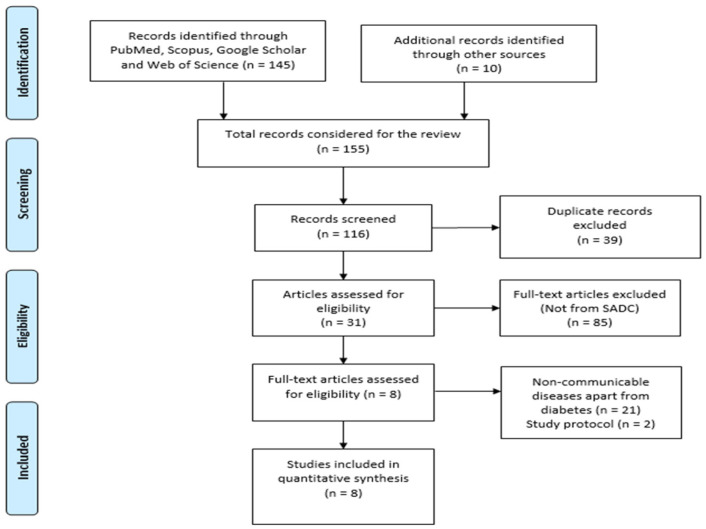
PRISMA flow chart of the selection process [17].

Search results were imported into Covidence Software (extraction template 2), a systematic review management tool, where duplicates were removed. Two independent reviewers screened titles and abstracts for relevance according to the predefined criteria. Full texts of potentially relevant articles were retrieved and assessed independently by the same reviewers. Disagreements were resolved through discussion or by consulting a third reviewer. The selection process is documented using a PRISMA flow diagram (Figure 1), indicating the number of studies identified, screened, included, and excluded, along with reasons for exclusion.

Reasons for removal of some articles: duplicate records (*n* = 39); articles not from SADC (*n* = 85); other non-communicable diseases (*n* = 21). Two of the articles were study protocols.

### 2.6. Data Extraction and Collection

A structured data extraction form was developed by the research team with the support of the faculty librarian at North-West University, Mafikeng, South Africa, and pilot-tested to ensure clarity and consistency. For each included study, the following data were extracted: author(s), year of publication, country, study design, setting, sample characteristics, description of the IPC intervention, health professionals involved, key barriers and facilitators reported, and outcomes related to implementation. Data extraction was conducted independently by two reviewers, AA & BB, and discrepancies were resolved through consensus with a third reviewer, UU.

### 2.7. Quality Assessment

The methodological quality of each included study was appraised using validated tools appropriate for its design. The Critical Appraisal Skills Programme (CASP) checklists were used for qualitative studies, the Joanna Briggs Institute (JBI) tool was used for quantitative studies, and the Mixed Methods Appraisal Tool (MMAT) was applied to mixed-methods studies [19,20]. Two reviewers independently conducted the appraisals and resolved any discrepancies through discussion. Quality ratings were used to interpret the robustness of the evidence but were not used as exclusion criteria.

### 2.8. Data Synthesis and Analysis

A narrative synthesis approach was used to analyze the data due to the heterogeneity of study designs and outcomes. Thematic analysis was employed to categorize reported facilitators and barriers. To structure the synthesis and interpret findings, the Consolidated Framework for Implementation Research (CFIR) was used. This framework categorizes implementation determinants into five domains: intervention characteristics, outer setting, inner setting, characteristics of individuals, and implementation process. Findings were thematically organized under these domains with slight modification to provide a structured comparison of factors across settings and contexts. Where appropriate, findings were stratified by country, care level (e.g., primary, secondary, tertiary), and type of IPC intervention. A summary table of included studies was also developed to highlight key characteristics and findings.

### 2.9. Ethical Considerations

This review involved the synthesis of published and publicly available data; therefore, ethical approval was not required. This systematic review was conducted in accordance with the Preferred Reporting Items for Systematic Reviews and Meta-Analyses (PRISMA) guidelines [17]. The methodological framework followed a structured approach to identify, screen, and synthesize evidence on the facilitators and barriers to effective use of IPC in the management of T2D in the elderly population of the SADC. The protocol was also registered with PROSPERO (available at https://www.crd.york.ac.uk/PROSPERO/view/CRD42024611402 (accessed on 7 November 2024).

## 3. Results

### 3.1. Participant Characteristics and Study Descriptions

Overall, most of the participants in the included studies were adults. Even though the focus of the review was on the elderly population, there are very few reports of studies in which the healthcare professionals worked collaboratively to care for only the elderly population in the region. This population included males and females, the majority of whom had T2D. The majority of the participants reported having other comorbidities. The description of each of the studies used in this systemic review is as presented in Table 2. In most of the articles, the healthcare team consisted of physicians, nurses, medical laboratory scientists, pharmacists, community health workers, and rural health motivators. These are professionals who, at different points in time and at different healthcare facilities, managed the elderly populations with diabetes in the Southern Africa Development Community (SADC). In this review, five of the studies were carried out in South Africa, one in Eswatini, one in Tanzania, and one in Zimbabwe. Most of the studies were conducted at primary healthcare facilities, indicating the preferred use of these facilities by the elderly diabetic patients in the region. In the articles reviewed, most of the studies made use of mixed study designs consisting of both qualitative and quantitative arms. Two studies had questionnaire-based designs. The study duration in the articles ranges from 3 months to 2 years.

The quality assessment tool used considers aspects such as the appropriateness of the research design, recruitment strategy, data collection, relationship between researchers and participants, ethical issues, data analysis, and clarity of findings.

### 3.2. Implementation Data

This review also evaluated some of the facilitators and barriers to implementing interprofessional care. Regarding the identified facilitators, Steyn et al. [21] suggested that consolidating relevant information about elderly patients with T2D into a single accessible document would streamline their treatment process. Meanwhile, Katz et al. [22] recommended providing decision support to primary healthcare nurses, scaling up medication, and ensuring access to specialist care. Piotie et al. [23] proposed that organizing training on clinical procedures, patient counseling, and mobile application use would enhance care delivery. Catley et al. [24] emphasized the importance of intensive training for healthcare workers, delivered in local languages. Furthermore, Sonday et al. [25] advocated for medication-based treatments as the primary focus in T2D management. Additionally, Munyogwa et al. [27] and Sharp et al. [28] identified formalized health education for patients during clinic visits and implementation of treatment guidelines as key approaches to facilitate IPC in T2D management in the region.

Regarding the barriers, Steyn et al. [21] noted that the substantial workload resulting from staff shortages, combined with the steady influx of new diabetes cases in the region, poses significant challenges to effective IPC implementation. Katz et al. [22] and Sharp et al. [28] identified patient loss to follow-up, fear of litigation, and policy restrictions on prescribing medications as major obstacles to effective T2D treatment. Piotie et al. [23] highlighted poor medical record keeping, lack of patient registries, and inadequate guidelines on task sharing as significant concerns. Catley et al. [24] found that enforcing dietary control was particularly challenging among patients. Sonday et al. [25] also noted low prescriber acceptance and clinical inertia, likely due to concerns about the potential consequences of prescriptions. Furthermore, Munyogwa et al. [26] reported that the absence of specific guidelines in certain situations hindered effective case management, underscoring the need for clearer guidance.

In most of the studies, the risk of bias was observed to be low.

The facilitators and barriers to implementing interprofessional care are presented in Table 3.

### 3.3. Intervention Details and Outcome Measures in the Selected Studies

Table 4 summarizes the intervention and outcome details in the selected studies. Different forms of interventions were used in the studies. These include differentiated service delivery models, a structured record that incorporated the National guidelines for the management of patients with diabetes in South Africa, a chronic disease outreach program, the Tshwane Insulin Project, and other video-based group sessions. There were no interventions at all in one of the studies. Most of the studies used the multidisciplinary team model. The role of each team member was defined in the majority of the studies following appropriate training of the team members. The majority of the studies had only an intervention group. The primary outcome measure used to assess the effectiveness of care in the studies was changes in the glycemic level. Some of the studies reported improvement in the glycemic level (or reduction in glycated hemoglobin level), while others reported no significant improvement. In most of the studies, the secondary outcomes of blood pressure, lipid profile, and body mass index were either not documented or not associated with significant improvement. Even though the majority of these studies are not clinical experimental studies where there were control groups for adequate comparison with experimental groups, the results point to slight improvement in diabetes management, which may be attributed to the cooperation among the healthcare team in providing care.

### 3.4. Quality Appraisal of Included Studies

A total of eight studies were appraised using three different critical appraisal tools, the CASP for qualitative studies, the Joanna Briggs Institute (JBI) tool for quantitative studies, and the Mixed Methods Appraisal Tool (MMAT) for mixed-methods research. The overall methodological quality across the studies ranged from moderate to high. See Table 5, Table 6 and Table 7 for a full description of the appraisal process. Among the qualitative studies appraised using the CASP tool, two were rated as high quality and three as moderate. All five studies [22,25,26,27,28] had clearly defined aims and used designs appropriate for qualitative inquiry. However, limitations were noted in areas such as the researcher–participant relationship, reflexivity, and the transparency of data analysis. For instance, while data collection methods were largely appropriate, three studies only partially addressed the influence of the researcher’s role on the findings. Ethical considerations were generally well addressed across studies.

For the quantitative studies, both articles [21,24] met all eleven JBI criteria, reflecting high quality in terms of sampling strategy, validity of measurement tools, appropriate statistical analysis, and response rate. These studies demonstrated rigorous methodological standards, especially in addressing confounding factors and using reliable and valid outcome measures. The single mixed-methods study by Piotie et al. [23], assessed using the MMAT, fulfilled all eight core criteria. The study had clearly defined research questions, used appropriate qualitative and quantitative approaches, and integrated both strands of data effectively. The interpretation of integrated findings was robust, and the study adhered to established quality criteria for both components. As such, the study was rated as high quality. The majority of the included studies demonstrated methodological rigor and transparency in reporting, enhancing the trustworthiness of the synthesized findings. Nonetheless, some moderate-quality studies revealed recurring methodological weaknesses, especially around reflexivity and analytic depth in qualitative work. These limitations should be considered when interpreting the broader evidence base. Two reviewers independently conducted the appraisals and resolved any discrepancies through discussion.

Table 5, Table 6 and Table 7 show the different tools for the quality appraisal of the included studies.

## 4. Discussion

This systematic review explored some of the facilitators and barriers to effective use of interprofessional care for elderly patients with diabetes mellitus in the SADC region. Given the scarcity of studies targeting this population, our review highlights the need for more research in this area. Most articles reviewed were qualitative, with interventions such as structured records, video-based group sessions, and outreach programs that may not directly impact primary outcomes. Notably, only one study, the Tshwane Insulin Project, was a clinical trial with a clear-cut intervention to reduce glycemic levels. Most of the studies had either non-medication-based interventions or no interventions at all. This approach might not be so efficient for elderly people in the SADC region, as the evidence from the studies may be prone to biases. In essence, attention should be focused on medication-based interventions for these older people. In addition to this, lifestyle modification, physical activity, regular screening for mood disorders, regular comprehensive geriatric assessments, and nutritional assessments are other modifications that should be considered for effective control of T2D in this category of patients. When managing diabetes in older adults, a multifaceted approach is crucial. Medication-based interventions should be a primary focus, taking into account the patient’s comorbidities, potential side effects, and medication adherence. In addition to pharmacological interventions, lifestyle modifications play a vital role. Encouraging physical activity, such as walking or swimming, can help improve insulin sensitivity and overall health. Regular screening for mood disorders, such as depression, is also essential, as mental health issues can impact diabetes management. Comprehensive geriatric assessments can help identify potential issues with cognitive function, mobility, and social support, allowing for targeted interventions. Nutritional assessments are also critical, as older adults with diabetes may have unique dietary needs. A balanced diet that takes into account the patient’s nutritional requirements, food preferences, and swallowing difficulties can help optimize glycemic control. By incorporating these modifications, healthcare providers can develop a personalized care plan that addresses the complex needs of older adults with diabetes, ultimately improving health outcomes and quality of life. A multidisciplinary team approach, involving healthcare professionals from various disciplines, can help ensure that these patients receive comprehensive and coordinated care.

The use of interprofessional care, a collaborative approach involving healthcare workers from different disciplines, has been proposed to strengthen outcomes. Interprofessional care is a collaborative clinical approach that allows healthcare workers from different disciplines to work together to provide patient-centered care [29]. This approach has been effective in managing hypertension and other non-communicable diseases, improving workforce engagement, patient outcomes, and cost-effectiveness in diabetes management [30]. Our review found that interprofessional care reduced healthcare facility congestion, patient waiting times, and treatment costs while improving health outcomes and risk factor control [31]. In this review, team work by the healthcare professionals made the healthcare facilities less congested, reduced patient waiting time, allowed patients to enjoy excellent communication with healthcare providers, improved health outcome and risk factor control, reduced the cost of treatment, and made arrangement for continuity of care possible.

Several facilitators to effective interprofessional care implementation were identified, including having relevant patient documents readily available, providing decision support to healthcare workers, and training of healthcare workers. Continuous engagement of these trained healthcare workers, use of local languages during the training sessions, and use of certified guidelines in the management of not only T2D but also all the other disease conditions were also crucial. These findings are supported by the report of Szafran et al. [32] and the Parliamentary Monitoring Group [33], which recommended the establishment of community-based services to provide self-management support, promote health, and ease access to medicine, as well as provide home visits by district-based community health workers, which could help overcome many of the barriers to care experienced by older patients [34]. Doing this will help to reduce waiting time, facility visits, and many other costs for the patients [35].

However, all these facilitators have not completely translated into better health outcomes, possibly due to the barriers in the implementation of interprofessional care. This is not unexpected as access to care remains a big challenge for the elderly due to physical disability, the cost of transport, childcare responsibilities, long waiting times, poor communication with healthcare providers, overcrowding, and fragmented care for different morbidities associated with old age [36]. Importantly, the good outcomes of interprofessional care may be masked by the enormous workload due to staff shortages, loss to follow-up, restriction of some health workers’ ability to take certain decisive actions, poor record keeping, and inefficient monitoring of patient compliance to prescribed dietary recommendations. Another study attributed the poor control of glycemia to the physician–patient ratio [37]. This review suggests that the current public healthcare system for older adults with T2D appears to be inefficient considering the reports of few significant improvements in the glycated hemoglobin level (primary outcome) and other markers of improved quality of life (secondary outcomes) like blood pressure, lipid profile, and body mass index. The fact that most of the studies were not clinical trials makes it easy to completely agree with this observation. Unfortunately, this supports the fact that in some parts of the SADC region, there appears to be poor service delivery at healthcare centers [33], which may be improved by a supportive, accessible healthcare system, especially at the primary care level [38]. In South Africa, for example, some community health centers are overcrowded and poorly resourced due to the multiple-disease burden, leaving limited time for front-line health workers to deal with the management of patients with diseases such as diabetes [39].

Another aspect to look at is the policy or regulatory guidelines for the management of diabetes in elderly patients in the region. Such guidelines are scarce, and the only South African document which specifically addresses the unique needs of the older diabetic patient is the 2017 Society for Endocrinology, Metabolism and Diabetes of South Africa (SEMDSA) guideline [40], which clearly states that the treatment goal in older adults should be glycemic control and a reduction in other risk factors for macrovascular and microvascular disease control for optimal disease management [41]. It has been reported that patients with diabetes in these health centers are ill-equipped to play active roles in self-care due to their limited opportunities for education and counseling [42,43]. In the context of the SADC, the facilitators and barriers to effective use of interprofessional care presented above point to the growing need to establish policies that promote task shifting where non-physicians are allowed to manage elderly T2D patients, regular training programs, role definitions, and improved communication [34,35].

Ultimately, what is necessary is an intervention tailored to the distinct needs of older adults with diabetes, thereby mitigating diabetes-related morbidity and associated healthcare expenditures [29,44]. To further validate this approach and for potential application in the SADC, the American Diabetes Association (ADA)’s Standards of Care in Diabetes-2025 emphasizes the importance of a multidisciplinary team establishing suitable goals, promoting dietary adjustments, advocating medication adherence, ensuring proper self-monitoring of blood glucose (SMBG), regularly assessing diabetes complications, and meticulously conducting laboratory evaluations [45]. Despite the challenges, interprofessional care has the potential to improve patient outcomes, and further research is needed to explore its effectiveness in the SADC region.

Contextual Considerations

The unique healthcare context of SADC countries influenced the quality of evidence. Resource limitations affected the implementation of interventions and the robustness of study designs. However, some studies [46,47] demonstrated commendable efforts in adapting rigorous research methods to resource-constrained settings.

Strengths of the Evidence Base

Despite the limitations, several strengths were noted in the evidence base including a well-designed RCT that provides a solid foundation for assessing the effectiveness of interprofessional care. Other studies employed innovative mixed-methods approaches, providing rich contextual data alongside the quantitative outcome, which enhanced the interpretability of findings in the SADC context.

Study limitations

This review has two major limitations. Firstly, only articles written in the English language were considered for the review. So, articles written in Arabic, French, and other languages that fulfilled the selection criteria were probably excluded. Future studies should therefore consider the use of multilingual databases to yield a more robust review. The language restriction in this review may have led to the exclusion of important studies published in non-English languages, potentially limiting the generalizability of the findings. This limitation highlights the need for future reviews to adopt a more inclusive approach, incorporating studies from diverse linguistic and cultural contexts. By doing so, researchers can provide a more nuanced understanding of the topic, capturing a broader range of perspectives and experiences. This, in turn, can inform more effective policies and interventions that cater to diverse populations and contexts. A multilingual approach can enhance the validity and relevance of the review findings. Also, few databases were used, and most are not based in Africa like African Journals Online (AJOL). A more robust outcome would be arrived at if more databases are used for future reviews. Common methodological weaknesses included short follow-up periods (the majority less than 12 months), which limited the assessment of long-term outcomes, and few studies on T2D in the elderly patients in the region. Most SADC countries were also underrepresented, potentially limiting the generalizability of findings across the entire SADC region.

Implications for Interpreting the Findings and Perspectives for Clinical Practice

The moderate-quality evidence for improved glycemic control suggests that IPC could be used to effectively manage T2D in elderly populations in SADC countries. However, the lower-quality evidence for other outcomes necessitates caution in interpreting these results. The findings provide a foundation for implementing interprofessional care models in older adults, but further high-quality research is needed, particularly for long-term outcomes and cost-effectiveness in various SADC contexts.

## 5. Conclusions

This systematic review identifies key facilitators and barriers to implementing effective interprofessional care for type 2 diabetes management in the elderly population of the Southern African Development Community (SADC). Understanding these factors can help healthcare professionals optimize their collaborative efforts, ultimately enhancing the quality of care and improving health outcomes for elderly patients with T2D in the region.

## Figures and Tables

**Table 1:** Inclusion and exclusion criteria.

S/N	Inclusion Criteria	Exclusion Criteria
1	Studies that evaluated the effectiveness of interprofessional care in diabetes mellitus in the elderly population. Studies including both younger and older adults also included.	Review papers and published protocols.
2	Full-text peer-reviewed articles (from 2000 to 2024).	Studies not peer-reviewed.
3	Studies conducted in the SADC countries.	Studies on infectious or communicable diseases.
4	Studies published in the English language.	Studies not published in the English language.
5		Studies that reported the effect of collaborative care on diabetes outside of the SADC.
6		Peer-reviewed articles in French, Arabic, and other African languages.
7		Studies performed in animals.
8		Abstracts of articles.
9		Books of proceedings.
10		Short communications.
11		Letters to editors.
		Gray literature and unpublished studies: due to quality concerns and possible lack of standardization in terms of the methodology, reporting, and quality.

**Table 2:** Participant characteristics and study descriptions.

Authors	Country	Number of Participants Enrolled	Age	Gender Distribution	Type of Diabetes	Young/Elderly	Setting	Data Collection Method and Study Design	Study Duration	Duration of Diabetes	Presence of Comorbidities
Steyn et al., 2013 [21]	South Africa	456	Mean age of 58.1 ± 10.9 years	24.5% males, 75.6% females	Majority type 2	Only elders	Community health centers (CHCs) in Cape Town	Questionnaire-based and simple experimental design (Quantitative)	9 months	Not stated	Obesity, Hypertension Hypercholesterolemia
Katz et al., 2009 [22]	South Africa	257	≥18 and ≤80 years old	39% males, 61% females	42% type 2	Young and elderly	Clinics and health centers in the South West Gauteng region	Questionnaire-based design (Qualitative)	2-year follow-up	Not stated	Obesity, Hypertension
Piotie et al., 2022 [23]	South Africa	36	Between 18 and 70 years	23.7% males, 76.3% females	All type 2	Young and elderly	Primary healthcare centers in the City of Tshwane, South Africa	Feasibility study; mixed (Qualitative and Quantitative)	7 months	Not stated	Hypertension
Catley et al., 2022 [24]	South Africa	494	Mean age of 68 years	Test group (28 males, 212 females)	All type 2	Elderly	Residents of a predominantly Xhosa-speaking urban township of Cape Town, South Africa	Cluster randomized controlled trial (Quantitative)	7 to 9 months	Not stated	Not stated
Sonday et al., 2022 [25]	South Africa	104	Mean age of 57.9 ± 9.2 years	32.7% males, 67.3% females	All type 2	Young and elderly	Community day center (CDC) in Cape Town, South Africa	Case study approach (Qualitative)	6 months	Not stated	Obesity, Hypertension Hypercholesterolemia
Frieden et al., 2020 [26]	Zimbabwe	188	Mean age of greater than 65 years	Not reported	Not stated	Elderly	Primary healthcare clinics (PHCs) and two hospitals in Chipinge district, Manicaland, in Zimbabwe	Descriptive study (Qualitative)	Not assessed	Not stated	Not stated
Munyogwa et al., 2020 [27]	Tanzania	330	Mean age of 40.27 ± 13.31 years	42.7% males, 57.3% females	All type 2	Young and elderly	Healthcare facilities in Mwanza Region, Tanzania	Cross-sectional study; analytic (Qualitative)	3 months	≤10 years	Obesity Hypertension
Sharp et al., 2020 [28]	Eswatini	68	Median age of 62 (range: 61–63) years	22% males, 78% females	All type 2	Majority elders	Clinics in the Lubombo Region of Eswatini	Observational study (Qualitative)	1 year 6 months	Not stated	Hypertension

**Table 3:** Implementation data of the included studies.

Authors	Risk of Bias Assessment	Facilitators to Implementing Interprofessional Care	Barriers to Implementing Interprofessional Care
Steyn et al., 2013 [21]	Some concerns	Having all the relevant information in one easily accessible document was a facilitator.	There was an enormous workload for the healthcare workers due to staff shortage and an increased influx of new diabetic patients at the community health centers.
Katz et al., 2009 [22]	Low risk	The primary healthcare nurses (PHCNs) were provided with decision support, escalated scaling up of medication, and prompt access to specialist care. Care was free in the primary care clinics and ranged from USD 0 to 8 at the specialist centers, depending on a patient’s employment status or age.	Loss to follow-up occurred. The nurses complained that their current skills were not recognized and acknowledged, and they remained concerned about litigation. Current protocols did not allow nurses to prescribe all DM medication. Staff shortages made it difficult to attend continuing education seminars, affecting PHCN access to information. In addition, nurses lacked the confidence and skills to initiate insulin dosing, and no clinic doctors were available to support decisions to initiate insulin or add new medications.
Piotie et al., 2022 [23]	Low risk	Healthcare providers attended a workshop on Integrated Diabetes Management in Primary Health Care. They also received training on the study procedures including sessions on the evidence and rationale for insulin therapy, patient counseling, initiation and titration of basal insulin, and the use of the mobile app. The community health workers were trained on what to do during home visits.	It was difficult for the team to identify eligible patients because of poor medical records and a lack of patient registries. Task sharing was a major challenge alongside the lack of involvement of allied healthcare workers.
Catley et al., 2022 [24]	Low risk	SMS content was adapted over time to refer to the content of the session that the participant had most recently attended. Intensive training was organized for the community health workers. The local Xhosa language was used for the training where applicable to ensure proper understanding.	It was difficult for the team to ensure a rigorous dietary intake measure that may have been related to HbA1c changes.
Sonday et al., 2022 [25]	Low risk	Pharmacological treatments were prescribed for stable patients with T2D at baseline.	The IPC team was faced with the challenge of low prescriber acceptance and clinical inertia from the patients. There was also the challenge of medication therapy problems like contraindication and medicine interactions which were not documented in some cases.
Frieden et al., 2020 [26]	Low risk	The IPC team developed simplified context-adapted clinical protocols, training materials, and patient literacy tools.	There were no NCD-specific guidelines to help the team.
Munyogwa et al., 2020 [27]	Low risk	Most of the respondents acknowledged receiving health education when first diagnosed (96.7%) and also during routine diabetes clinic visits (97.0%).	The team was overworked due to a large number of patients.
Sharp et al., 2020 [28]	Low risk	The team developed a treatment guideline in the form of a desk guide for cardiovascular disease, diabetes, and hypertension, which dealt with the following: primary prevention, identification and screening, diagnosis, education, treatment, and referral.	The team was faced with the challenge of loss to follow-up. There was also the challenge of consistent rotation of nursing staff.

**Table 4:** Intervention and outcome details in the selected studies.

Authors	Tool for the IPC Sessions	IPC Model	Composition of the IPC Team	Frequency and Duration of IPC Sessions	Specific Roles of Each Team Member	Any Training Provided to the IPC Team	Primary Outcome	Secondary Outcomes			
Steyn et al., 2013 [21]	A structured record (SR) which incorporated the National guidelines for the management of patients with diabetes or hypertension or both conditions was designed and used	Multidisciplinary teams	Physicians, nurses	Not reported	Nurses administered the SR. Patients were referred to physicians for further treatment.	The nurses were trained.	Changes in glycemic control (HbA1c levels)	Blood pressure control	Lipid profile changes	Body mass index (BMI)	Other outcomes like proportions of patients with recorded examinations for complications (retinopathy, nephropathy, foot problems). Number of patient folders that contained SR and degree to which SR was completed also examined
Katz et al., 2009 [22]	A chronic disease outreach program (CDOP) based on the chronic care model was used	Integrated delivery of PHC services (chronic care model)	Nurses, physician (Nephrologist)	48 clinic visits over 2 year-period	The health workers evaluated ‘functional’ clinical outcomes. They administered a questionnaire assessing nurses’ knowledge, their education support, and the value of the CDOP.	The health workers were trained.	Intervention lacked required effect on diabetes, degree of glycemic control poor	No improvement in BP control	No significant change	No significant change	No significant change in creatinine level
Piotie et al., 2022 [23]	The Tshwane Insulin Project (TIP) involved initiation of basal insulin (Protophane Humulin N [NPH]), which was nurse-led, assisted remotely by a doctor	Multidisciplinary teams	Nurses, physicians, community health workers	5 clinic visits, 14 weekly home visits and 13 post-insulin initiation follow-up visits	The health workers administered the insulin therapy and initiated the use of the mobile app.	The participating healthcare providers attended a workshop on Integrated Diabetes Management in Primary Health Care. They also received training on the study procedures including sessions on the evidence and rationale for insulin therapy, patient counseling, initiation and titration of basal insulin, and the use of the mobile app.	Level of DM control poor overall, with only 31% of patients having optimal control at HbA1c <7%, and mean HbA1c of 9 ± 3%	Only baseline values reported	Men more likely than women to have higher cholesterol	BMI patterns similar, although women more likely to be obese [(BMI 30–39.9 kg/m^2^), (M:F; 28%:47%)] or morbidly obese [(BMI >40 kg/m^2^), (M:F; 7%:18%)]	Improved early detection and referral of high-risk patients. Sensitivity for detecting those needing referral of 95%, and specificity for those not requiring referral of 100%, i.e., positive predictive value for appropriate referral of 100%
Catley et al., 2022 [24]	The tools included 17 video-based group sessions and measurement of some clinical parameters	Multidisciplinary teams	Community health workers, clinicians	Sessions delivered weekly	Community health workers (CHWs) handled the sessions. Clinicians trained the CHWs.	CHWs received basic training in in-home-based care, chronic disease management, and wellness, and received training in nutrition and counseling as well	Slight glycemic control recorded with 2.2% lowering in HbA1c values	Not reported	Not reported	Not reported	Healthcare professionals satisfied with initiation, application, and appropriateness of intervention
Sonday et al., 2022 [25]	Prescribed medication therapy management led by a pharmacist was used	Integrated team care	Pharmacists, health promoters, lab scientists, nurses, physicians	Weekly medication therapy	Pharmacists worked on the patients’ folders.	The researcher, a pharmacist trained in pharmacotherapeutics, attended a 1-year course, ‘Integrated applied therapeutics: Fundamentals of rational prescribing’ (2015), offered by Pharmacy Education International, an approved South African Pharmacy Council provider.	Significant reduction in HbA1c levels	No significant change	No significant change	No significant change	7% weight loss and 150 min/week of physical activities reported
Frieden et al., 2020 [26]	Free medications subsidized by MSF Advocacy were used to improve MoH medication supply to health facilities	Nurse-led differentiated care	Nurses, pharmacy technicians, doctors	Monthly	Doctors managed critical cases referred by the nurses.	The MSF mentoring team provided structured teaching sessions and hands-on clinical training to MOH staff, who performed consultations. The mentoring curriculum comprised clinical and programmatic knowledge. On-the-job support was provided by the mentor. A competence dashboard was used to monitor progress.	Optimal glycemic control (HbA1c < 7%) only achieved in 8.6% (2015) and 11.5% (2016) of patients	No significant change	No significant change	Not documented	Large number of medication therapy problems identified
Munyogwa et al., 2020 [27]	A structured questionnaire was used	Integrated team care	Nurse, physicians, medical officers, nutritionist /dietitian	3 months	Health workers administered questionnaires and measured selected parameters.	Health workers were trained properly.	Not reported	Not reported	Not reported	Not reported	Free medications improving access to care for patients; Reliable Médecins Sans Frontières (MSF)-supported supply allowing spacing of patient appointments
Sharp et al., 2020 [28]	This was a nurse-led pilot implementation study	Multidisciplinary teams	Nurses, pharmacists, doctor	18 months	Health workers administered questionnaires and measured selected parameters.	The nurse-led team developed and administered a 3.5-day skill-based and interactive training program for clinic nurses, which covered all elements of the desk guide including data collection, and was based around a combination of lectures, demonstrations, and interactive case study-based clinical role-play.	Only baseline values reported	Only baseline values reported	Only baseline values reported	Only baseline values reported	Blood pressure, random blood glucose, and body weight and height measurements performed

**Table 5:** CASP appraisal summary with overall quality.

Authors (Year)	Clear Aims	Appropriate Methodology	Design Fit for Purpose	Recruitment Strategy	Data Collection	Researcher–Participant Relationship	Ethical Considerations	Rigorous Analysis	Clarity of Findings	Value of Research	Overall Quality
Katz et al. [22]	Yes	Yes	Yes	Partial	Yes	Partial	Yes	Partial	Yes	High	Moderate
Sharp et al. [28]	Yes	Partial	Yes	Yes	Yes	No	Yes	Partial	Yes	High	Moderate
Freiden et al. [26]	Yes	Yes	Yes	Yes	Yes	Partial	Yes	Yes	Yes	High	High
Moyungwa et al. [27]	Yes	Yes	Yes	Yes	Yes	Partial	Yes	Yes	Yes	High	High
Sonday et al. [25]	Yes	Partial	Yes	Yes	Yes	Not Applicable	Not Applicable	Yes	Yes	High	Moderate

**Table 6:** JBI appraisal summary for quantitative studies.

Authors (Year)	Appropriate Sample Frame	Appropriate Sampling Method	Adequate Sample Size	Study Subjects and Setting Described	Valid Methods for Condition	Standard, Reliable Measurements	Appropriate Statistical Analysis	Identified Confounding Factors	Strategies for Confounding Factors	Valid and Reliable Outcomes Measured	Response Rate Adequate	Overall Quality
Steyn et al. [21]	Yes	Yes	Yes	Yes	Yes	Yes	Yes	Yes	Yes	Yes	Yes	High
Catley et al. [24]	Yes	Yes	Yes	Yes	Yes	Yes	Yes	Yes	Yes	Yes	Yes	High

**Table 7:** MMAT appraisal summary for mixed-methods studies.

Authors (Year)	Clear Research Questions	Data Collected Address Questions	Appropriate Qualitative Approach	Appropriate Quantitative Approach	Integration of Qualitative and Quantitative Data	Interpretation of Integration	Handling of Inconsistencies	Quality Criteria Adhered to	Overall Quality
Piotie et al. [23]	Yes	Yes	Yes	Yes	Yes	Yes	Yes	Yes	High

## Data Availability

The data used for this review is readily available upon a reasonable request.

## Abbreviations

The following abbreviations are used in this manuscript:

SADC	Southern Africa Development Community.
DM	Diabetes Mellitus
PRISMA	Preferred Reporting Items for Systematic reviews and Meta-Analyses
PROSPERO	International Prospective Register of Systematic Reviews
NCDs	Non-Communicable Diseases
PICO	Population, Intervention, Comparison, and Outcome

## Appendix A

### Search Process of Pubmed for the Study

1. Search terms were clearly defined using some important keywords in the topic e.g.,: “Diabetes Mellitus Type 2 (T2D)”, “Elderly Population”, “Interprofessional Care”, “Systematic Review”, “Southern Africa Development Community (SADC)”.
2. Search query was conducted using Boolean operators e.g.,: (“Diabetes Mellitus Type 2 (T2D)” OR “T2D” OR “Non-insulin-dependent diabetes mellitus (NIDDM)” OR “Adult-onset diabetes”) AND (“Elderly Population” OR “Adults” OR “Older Adults”) AND (“Interprofessional Care” OR “Collaborative care” OR “Multidisciplinary care” OR “Integrated care” OR “Patient-centered care”).
3. The results obtained were filtered using language (English), date (2000 to 2025), design (qualitative, quantitative or mixed).
4. The search was then executed.
